# Supplementary material for: A pathogen-induced putative NAC transcription factor mediates leaf rust resistance in barley
Source: Nat Commun. 2023 Sep 6;14:5468. doi: 10.1038/s41467-023-41021-2 (PMC10482968; doi:10.1038/s41467-023-41021-2)
Supplement: Supplementary file 1 — Supplementary Information [file 41467_2023_41021_MOESM1_ESM.pdf]

A pathogen-induced NAC transcription factor mediates leaf rust resistance in barley

Chen *et al*.

Supplementary Table 1. Recombination-based fine mapping using four F_3 families from the Cebada Capa x WABAR2722 mapping population.

Recombinant	Phenotype		Markers					
ID*	F2	F3	S1534	BC00652	PK3	PG1	PG4	HGA1
59	;1-CN	Segregating (Seg)	HR	HR	Seg	Seg	Seg	Seg
92	;12CN	Seg	HR	HR	Seg	Seg	Seg	Seg
118	;1-CN	Seg	Seg	Seg	Seg	Seg	Seg	HR
145	:1-CN	Homozygous Resistance (HR)	HR	HR	HR	HR	Seg	Seg

***** 16 plants per F_3 family have been individually phenotyped and genotyped. The phenotypes and genotypes of the F3 families were either segregating (seg) or homozygous resistant (HR) for the *Rph7* phenotype and marker genotypes of the *Rph7* carrying parent Cebada Capa parent respectively.

Supplementary Table 2. Annotation of BAC AY642926 using FGenesh.

ID	Conserved domain class	Expressed	Gene	Start*	Stop	Strand
FGENESH_1	PTZ00395	yes	*UnkP-1*	4952	5431	-
FGENESH_2	unknown	no		8698	10783	-
FGENESH_3	unknown	no		18796	20067	-
FGENESH_4	ZnF-BED1	yes	*ZnF-BED1 (Rph7)*	22759	23955	-
FGENESH_5	unknown	no		25402	31265	-
FGENESH_6	transposon elements	no		34366	39278	-
FGENESH_7	PTZ00395	yes	*UnkP-2*	52766	53245	+
FGENESH_8	transposon elements	no		58357	66088	-
FGENESH_9	ZnF-BED2	yes	*ZnF-BED2*	70348	71610	-
FGENESH_10	GRDP-like	yes	*HvPG1*	75101	79806	-
FGENESH_11	transposon elements	no		84330	87906	+
FGENESH_12	transposon elements	no		92748	96728	+
FGENESH_13	transposon elements	no		98544	105793	+
FGENESH_14	DUF4371 superfamily/CLTH/LisH	yes	*HvPG4*	107654	114129	-
FGENESH_15	DUF563	yes	*HVHGA2*	119398	122419	-
FGENESH_16	SMC_prok_A superfamily	no		125127	130378	+
FGENESH_17	transposon elements	no		131716	135961	-
FGENESH_18	unknown	no		140860	141471	-
FGENESH_19	DUF563	yes	*HvHGA1*	144762	148016	-
FGENESH_20	FHY3 superfamily	no		149426	153378	+
FGENESH_21	Peptidase_C48 superfamily	no		154210	162024	-
FGENESH_22	BfiI_C_EcoRII_N_B3 superfamily	no		165581	167380	-
FGENESH_23	transposon elements	no		167995	176129	+

*Coordinates based on AY642926.1 of Scherrer *et al.* [29]

Supplementary Table 3. Details of five Australian pathotypes of *Puccinia hordei* used in the present study.

Pathotype	Culture number*	Year of collection	Virulence**
200 P+***	570	1990	*Rph8, Rph19*
276 P+	675	2017	*Rp2, Rph3, Rph4, Rph5, Rph6, Rph8, Rph19*
5457 P+	612	2009	*Rph1, Rph2, Rph3, Rph4, Rph6, Rph9, Rph10, Rph12, Rph19*
5477 P-	672	2017	*Rph1, Rph2, Rph3, Rph4, Rph5, Rph6, Rph9, Rph10, Rph12*
5553 P+	691	2020	*Rph1, Rph2, Rph4, Rph6, Rph7, Rph9, Rph10, Rph12, Rph19*

*Plant Breeding Institute culture number

**Tested for virulence on resistance genes *Rph1, Rph2, Rph3, Rph4, Rph5, Rph6, Rph7, Rph8, Rph9, Rph10, Rph11, Rph12, Rph13, Rph14, Rph15, Rph17, Rph18, Rph19*

***Pathogenicity for *Rph6* of isolates avirulent on *Rph2* could not be assigned due to the presence of both *Rph2* and *Rph6* in the differential line Bolivia

Supplementary Table 4. Complementation experimental data summarising phenotypic and genotypic assessment of Golden Promise + *Rph7* T_1 generation families at the seedling stage.

		B114-1		B114-2		B114-3		B114-17		B114-18	
**Pathotype**	**Plant**	**Phenotype****	**Genotype**	**Phenotype**	**Genotype**	**Phenotype**	**Genotype**	**Phenotype**	**Genotype**	**Phenotype**	**Genotype**
**5477 P-**	1	R	+	R	+	S	-	R	+	R	+
	2	R	+	R	+	S	-	R	+	R	+
	3	R	+	R	+	S	-	R	+	R	+
	4	R	+	R	+	S	-	R	+	R	+
	5	R	+	S	-	S	-	R	+	R	+
	6	R	+					S	-	R	+
	7	S	-					S	-	R	+
	8	S	-					S	-		
**276P+**	9	R	+	R	+	S	-	R	+	R	+
	10	R	+	R	+	S	-	R	+	R	+
	11	R	+	R	+	S	-	R	+	R	+
	12	R	+	R	+	S	-	R	+	R	+
	13	R	+	R	+	S	-	R	+	R	+
	14	R	+	R	+	S	-	R	+	R	+
	15	S	-	S	-			S	-	S	-
	16			S	-			S	-		
**200P+**	17	R	+	R	+	S	-	R	+	R	+
	18	R	+	R	+	S	-	R	+	R	+
	19	R	+	R	+	S	-	R	+	R	+
	20	R	+	R	+	S	-	R	+	S	-
	21	R	+	R	+	S	-	R	+	S	-
	22	S	-	S	-	S	-	R	+		
	23	S	-	S	-	S	-	S	-		
	24					S	-				
**5553P+**	25	S	+	S	+	S	-	S	+	S	+
	26	S	-	S	+	S	-	S	+	S	+
	27	S	+	S	-	S	-	S	-	S	+
	28	S	+	S	+	S	-	S	+	S	+
	29	S	+	S	+	S	-	S	-	S	-
	30	S	+	S	-	S	-	S	+	S	-
	31	S	-	S	+			S	-	S	+
	32	S	+	S	+			S	+	S	+
			23/7		21/7				21/10		22/5
**Controls**											
Golden Promise		S	-								
Golden Promise		S	-								
BW758		R	+								
BW758		R	+								
Cebada Capa		R	+								
Cebada Capa		R	+								
Galaxy		R	+								
Galaxy		R	+								

*T_1 sib plants were phenotyped as either resistant (R) or susceptible (S) using a qualitative scale therefore the semi-dominance of the *Rph7* resistance cannot be determined in transgenic families.

Supplementary Table 5. Top five unique protein structures obtained from the protein databank following a structure-structure search using the *Rph7* AlphaFold2 prediction.

PDB ID	Z-score	RMSD	Length aligned	Sequence identity (%)	PDB description
1ut4	9.2	2.6	83	23	DNA-binding NAC domain of Arabidopsis ANAC
3ulx	7.9	3	86	20	Rice Stress-responsive NAC1
3h1z	5.1	2.8	61	3	AP-2 COMPLEX SUBUNIT BETA-1
6e8a	5	3.1	64	8	DcrB from *Salmonella enterica*
6yai	5	2.8	61	3	Clathrin heavy chain

Supplementary Table 6. Samples processed for RNA-Seq.

ID	Timepoint	Treatment	Sample
B_1	day_1	mock_oil	BW758*
B_2	day_1	mock_oil	BW758
B_3	day_1	mock_oil	BW758
B_4	day_1	mock_oil	Cebada Capa
B_5	day_1	mock_oil	Cebada Capa
B_6	day_1	mock_oil	Cebada Capa
B_7	day_1	mock_oil	Bowman
B_8	day_1	mock_oil	Bowman
B_9	day_1	mock_oil	Bowman
B_24	day_1	infected_5457P+	BW758
B_25	day_1	infected_5457P+	BW758
B_26	day_1	infected_5457P+	BW758
B_27	day_1	infected_5457P+	Cebada Capa
B_28	day_1	infected_5457P+	Cebada Capa
B_29	day_1	infected_5457P+	Cebada Capa
B_30	day_1	infected_5457P+	Bowman
B_31	day_1	infected_5457P+	Bowman
B_32	day_1	infected_5457P+	Bowman
C_1	day_6	mock_oil	BW758
C_2	day_6	mock_oil	BW758
C_3	day_6	mock_oil	BW758
C_4	day_6	mock_oil	Cebada Capa
C_5	day_6	mock_oil	Cebada Capa
C_6	day_6	mock_oil	Cebada Capa
C_7	day_6	mock_oil	Bowman
C_8	day_6	mock_oil	Bowman
C_9	day_6	mock_oil	Bowman
C_25	day_6	infected_5457P+	BW758
C_26	day_6	infected_5457P+	BW758
C_27	day_6	infected_5457P+	BW758
C_28	day_6	infected_5457P+	Cebada Capa
C_29	day_6	infected_5457P+	Cebada Capa
C_30	day_6	infected_5457P+	Cebada Capa
C_31	day_6	infected_5457P+	Bowman
C_32	day_6	infected_5457P+	Bowman
C_33	day_6	infected_5457P+	Bowman

*BW758 refers to the near isogenic line Bowman+*Rph7*

Supplementary Table 7. Oligo primer sequences used in the study.

Primer name	Primer sequence (5'→3')	Use
Rph7C1F1	AAAGCGGCCGCACGATTATCATGAACAAAGAAATATAATAATAACTAATT	cloning
Rph7C1F2	AAAGCGGCCGCAGAGAAAGCTCATGCGGGGAG	cloning
Rph7C1R1	AAAGCGGCCGCGGCAATTCTTGCCTGACAGTGAT	cloning
Rph7C1R2	AAAGCGGCCGCTCCGCCGAAGCCCCTAAATG	cloning
s1543 F1	CGCCTTTTCCTTATCTCCCA	mapping
s1543 R1	CATGAGAAATTTGATTTGACCGA	mapping
BC00651F	TGCCATTGCCAGCCTAGAAA	mapping
BC00652R	GAGTTGCTCCCGCGAAATCT	mapping
M1-1434 F1	TAAACTGCTCCATGGGGAGG	mapping
M1-1434 R1	CCAATCCGGCATTACTTGCT	mapping
M1-0112 F1	CTACTCGTCGGAATTCATTTCTTTG	mapping
M1-0112 R1	CCGAGGACCGTTACCGAA	mapping
M1-0886 F1	ACTGGGAACATTGGAAACTGC	mapping
M1-0886 R1	AACTGATTGGTGTCAAGGATCGT	mapping
HvPG4KASPFAM3	GAAGGTGACCAAGTTCATGCTGTCATGCTCAAAACAGGTAACCCT	mapping
HvPG4KASPVIC3	GAAGGTCGGAGTCAACGGATTGTCATGCTCAAAACAGGTAACCCA	mapping
HvPG4 KASP R3	GTATCCCTACGCAGTTATTTGACCTATG	mapping
HvPK3KASPFAM5	GAAGGTGACCAAGTTCATGCTTCTACCACCCGGCTGCCA	mapping
HvPK3KASPVIC5	GAAGGTCGGAGTCAACGGATTTCTACCACCCGGCTGCCC	mapping
HvPK3KASPR5	GTCGAGGGGTGTGGACCA	mapping
HvPG1 new F1	TCCAGGTGTCAGGATCTATTCGT	mapping
HvPG1 new R1	GCCGGTCGATGATATTTGTGTA	mapping
HvHGA1 F2	AGCTTTCTTTCCCGCGTC	mapping
HvHGA1 R2	TAATCACACTTCGAAGCTGCTG	mapping
Rph7_RT_F2	ACACGATGTTGAAGTTAAAAGGTGG	qRT-PCR
Rph7_RT_R1	ACCATTTTTTTCGGCGACCAA	qRT-PCR
Rph7_RT_R2	AGAGGCACCGTTGAAGAGTTG	qRT-PCR, genotyping
Rph7_RT_F1	GAGAAAAACGCTCTTACTCGACC	qRT-PCR, genotyping
Rph7C1_seqR2	ACCGTCCACCGCAGTCGT	sequencing
Rph7C1_seqF2	AGGTTCGCACATTCACCCTC	sequencing
Rph7seqR2	CTCCTTCTCCCCAATCCCCATA	sequencing
Rph7seqF1	ACTTACTGGTGGCAGTGGTGG	sequencing
Rph7f1	GAGTAGAGCAGTCCCAGGCC	sequencing
Rph7seqF2	CTTGCAGCGGGTAGGTGTGG	sequencing
Rph7seqR1	CCACACCTACCCGCTGCAAG	sequencing
Rph7_attB1	GGGGACAAGTTTGTACAAAAAAGCAGGCTTACTGCATCATCAATCTCCACGGT	cloning
Rph7_attB2	GGGGACCACTTTGTACAAGAAAGCTGGGTCTGAGTAGAGCAGTCCCAGGC	cloning
Rph7_ATG attB1	GGGGACAAGTTTGTACAAAAAAGCAGGCTTAATGGCAAGCATCGGTGAGATC	cloning
Rph7_END attB2	GGGACCACTTTGTACAAGAAAGCTGGGTCCACCAGCTCTAGTTGTTCGGC	cloning

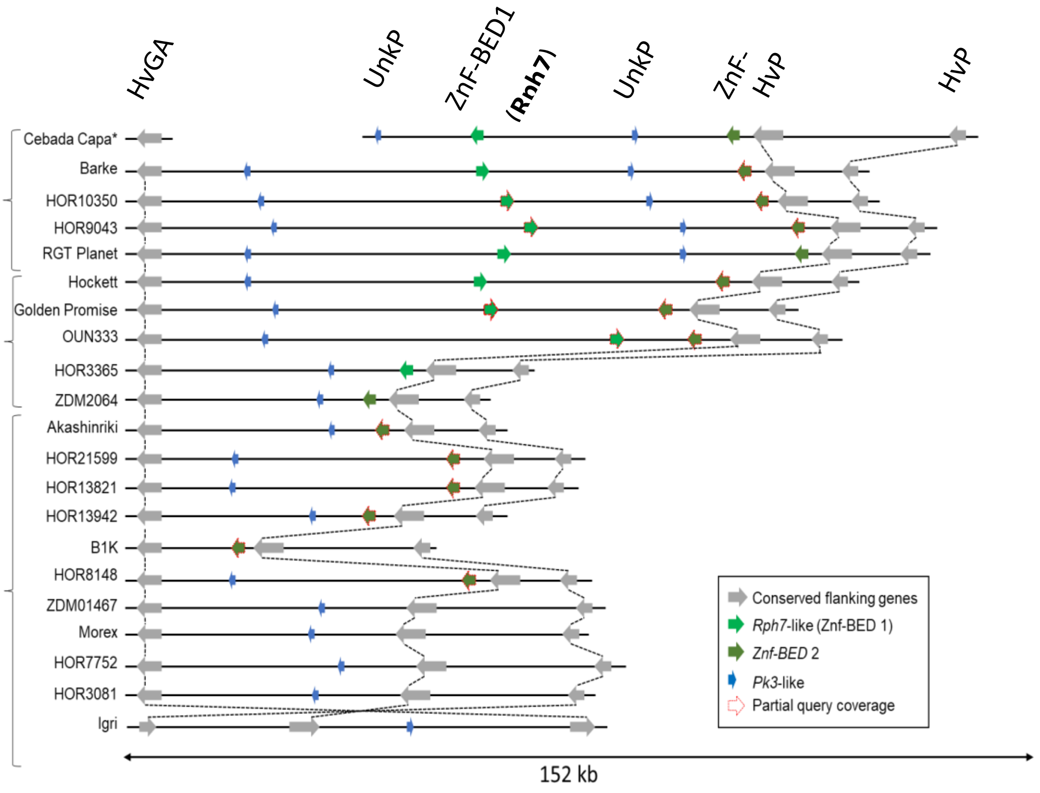

Supplementary Figure 1. Pan-Genomic representation of expressed genes detected within the genetic interval between *HvGAD1* and *HvPG4* at the *Rph7* locus. Haplotype I (HI) show the highest level of structural conservation to the *Rph7* resistance haplotype in Cebada Capa evidenced by size and gene content. HII accessions carry a homolog of *Rph7* but are missing either one or more of the other expressed genes within the interval. HIII accessions have no homologous sequence to *Rph7*, or the other Cebada Capa expressed genes, which is evidenced by a reduced interval size indicative of a presence/absence variation. Source data are provided as a Source Data file.

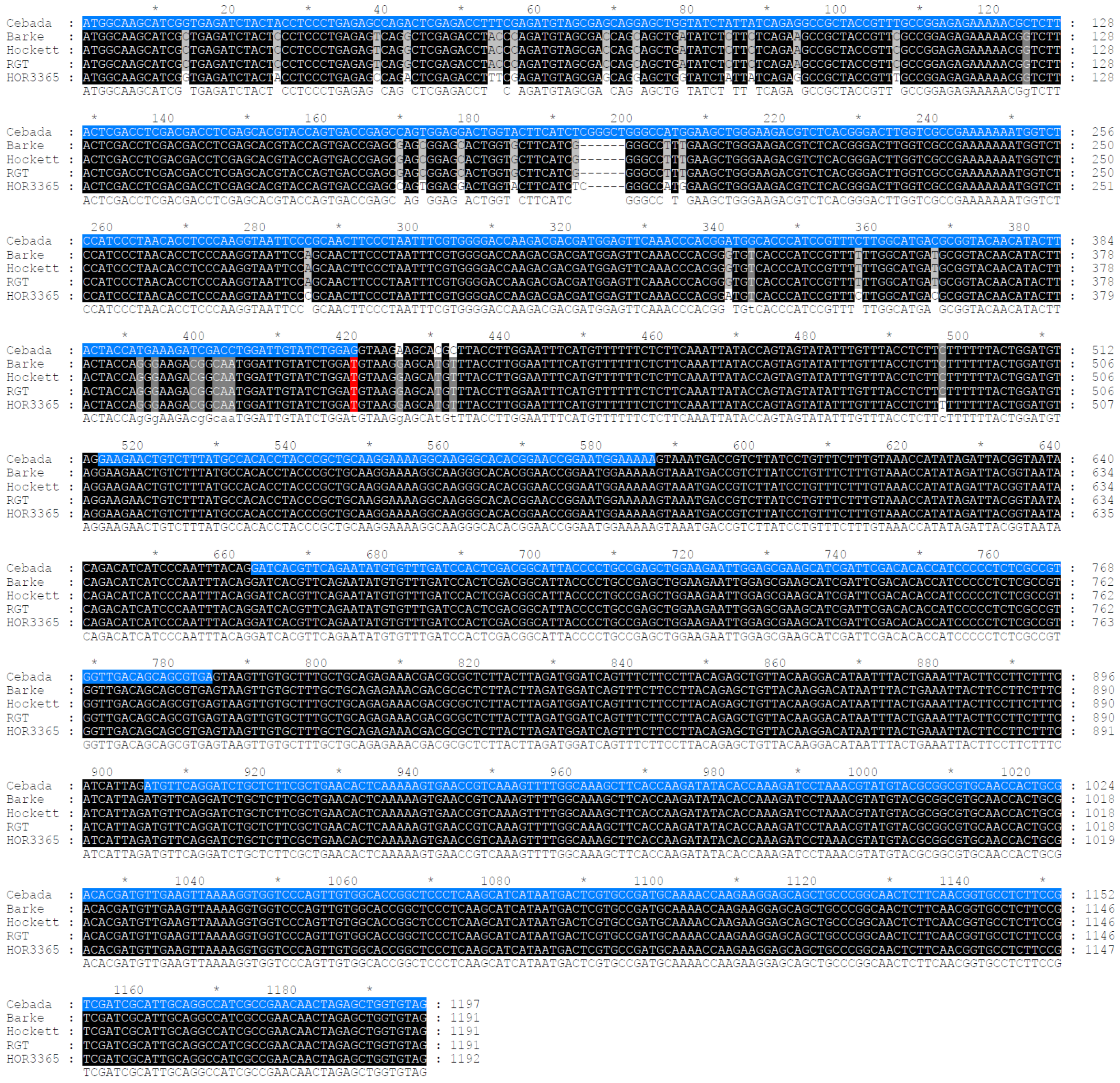

Supplementary Figure 2. Sequence alignment of genomic sequence of the full-length *Rph7* homologs detected in the barley pan-genome. The top row represents the full-length genomic sequence of the *Rph7* candidate gene *ZnF-BED1* in resistant donor cultivar Cebada Capa with exons highlighted in blue. Sequence variations are restricted to the N-terminal part of the gene within the predicted NAC domain. The homolog sequences carry a SNP mutation at the end of Exon 1 (highlighted in red) which likely causes an alternated splicing variant and does not translate to a full-length protein with similarity to *Rph7*. Putative translation stop sites are highlighted by dashed red rectangle. The remaining four partial homologs (in accessions HOR10350, HOR9043, Golden Promise and OUN333) cover the last 793 bp conserved with the displayed sequences and are not shown in this supplementary figure.

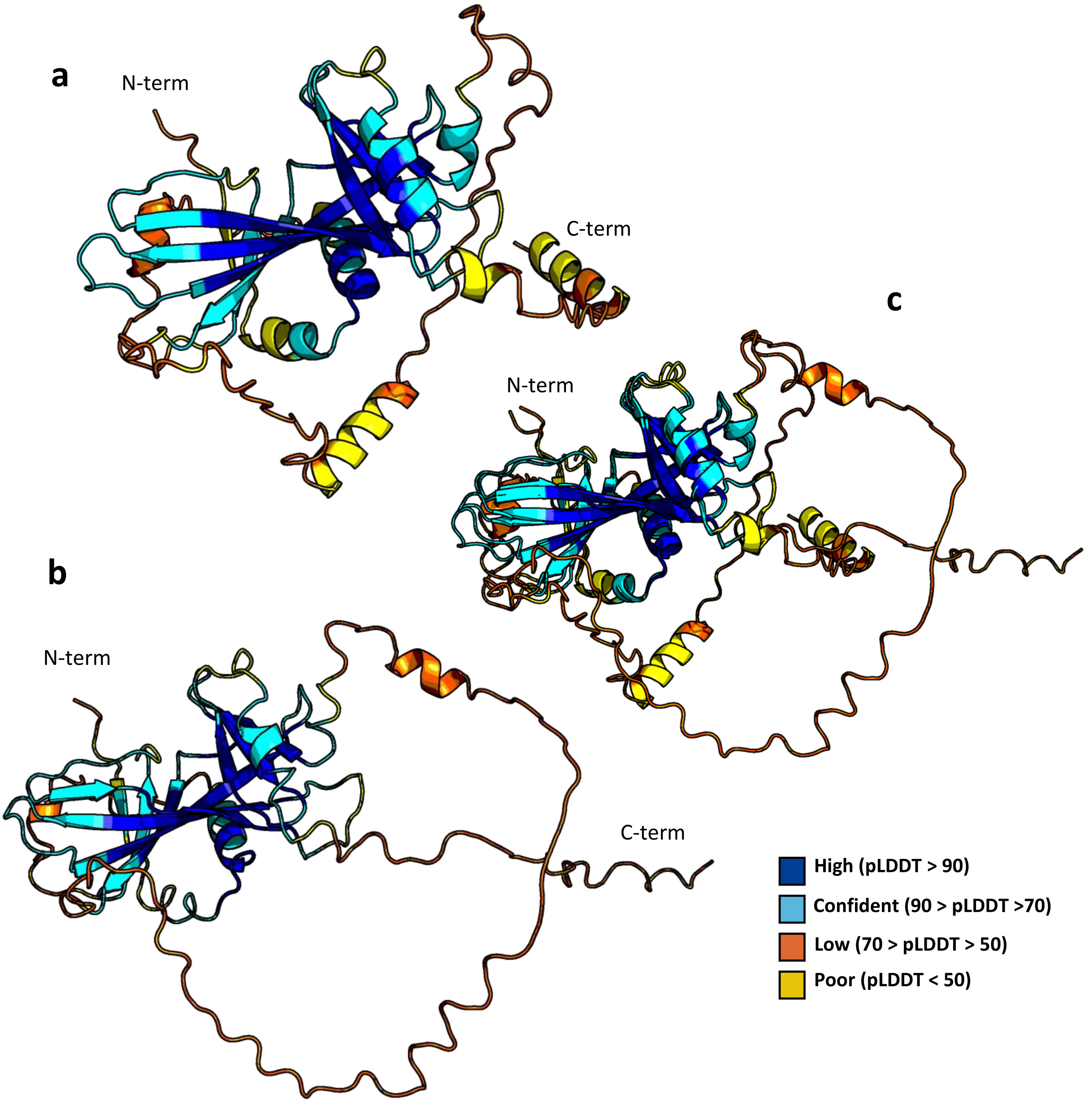

Supplementary Figure 3. Rph7structure prediction using AlphaFold2. a The top ranked model produced by Alphafold2 for Rph7, shown in cartoon representation and coloured according to confidence score (pLDDT), as depicted. where red is the most confident and blue is the least confident. **b** The top ranked model of Rph7 predicted using AlphaFold2 in the absence of templates to reduce model bias. Colouring is represented as in A. **c** Structural superimposition of the two predicted Rph7 structures using the Dali server pairwise alignment function. The predicted structures root mean-squared deviation of 1.2 Å across the entire protein.

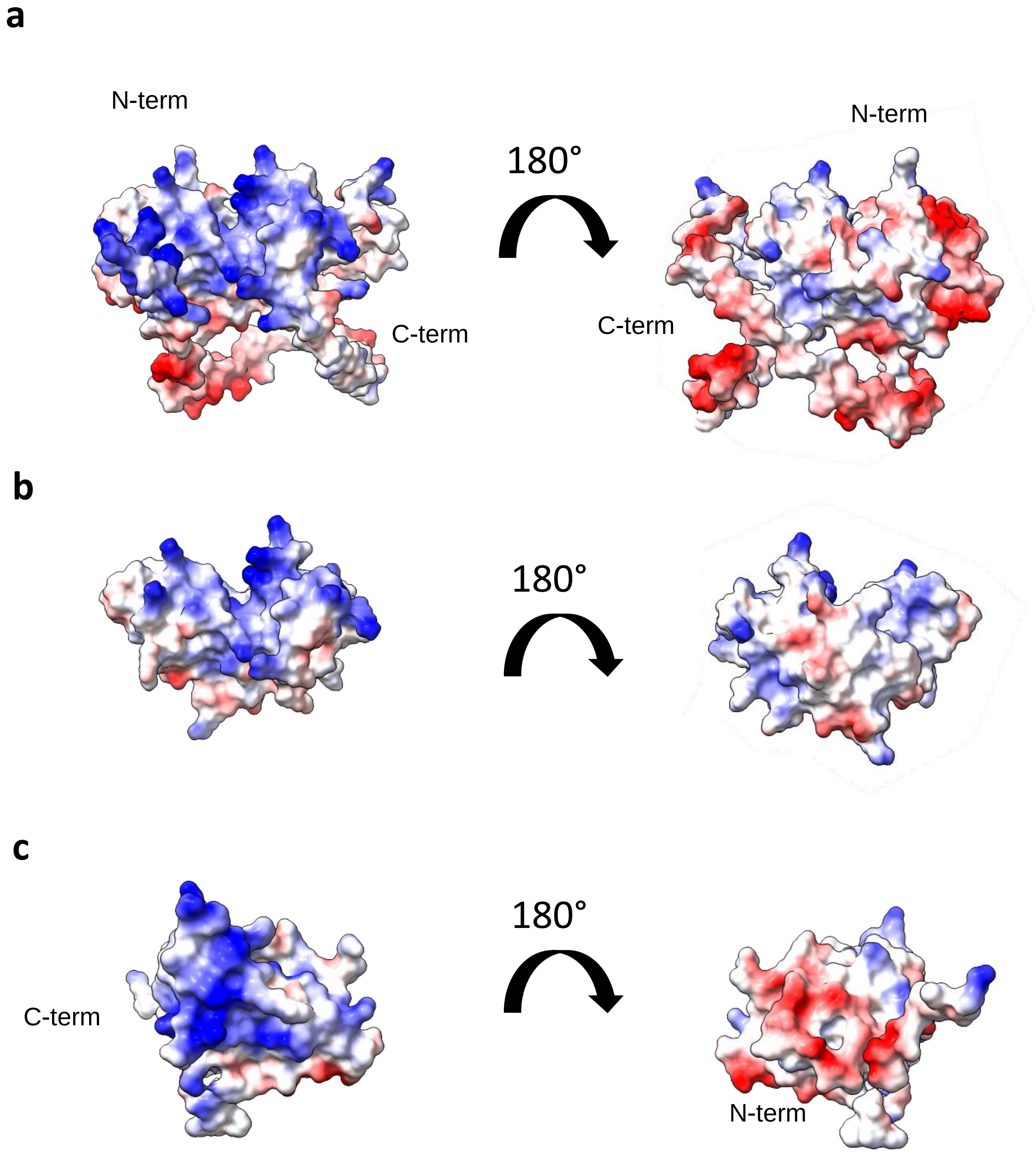

Supplementary Figure 4. ZnF-BED1 has a large positively charged surface patch suggesting it may bind DNA. Surface representation of the AlphaFold prediction of **a** ZnF-BED1, **b** ZnF-BED highly confident regions (pLDDT score >70) only, **c** and Arabidopsis ANAC019 (1ut4) showing Coulombic electrostatic potential generated with ChimeraX. The surface is coloured red and blue for negative and positive charges, respectively, and white represents neutral residues.

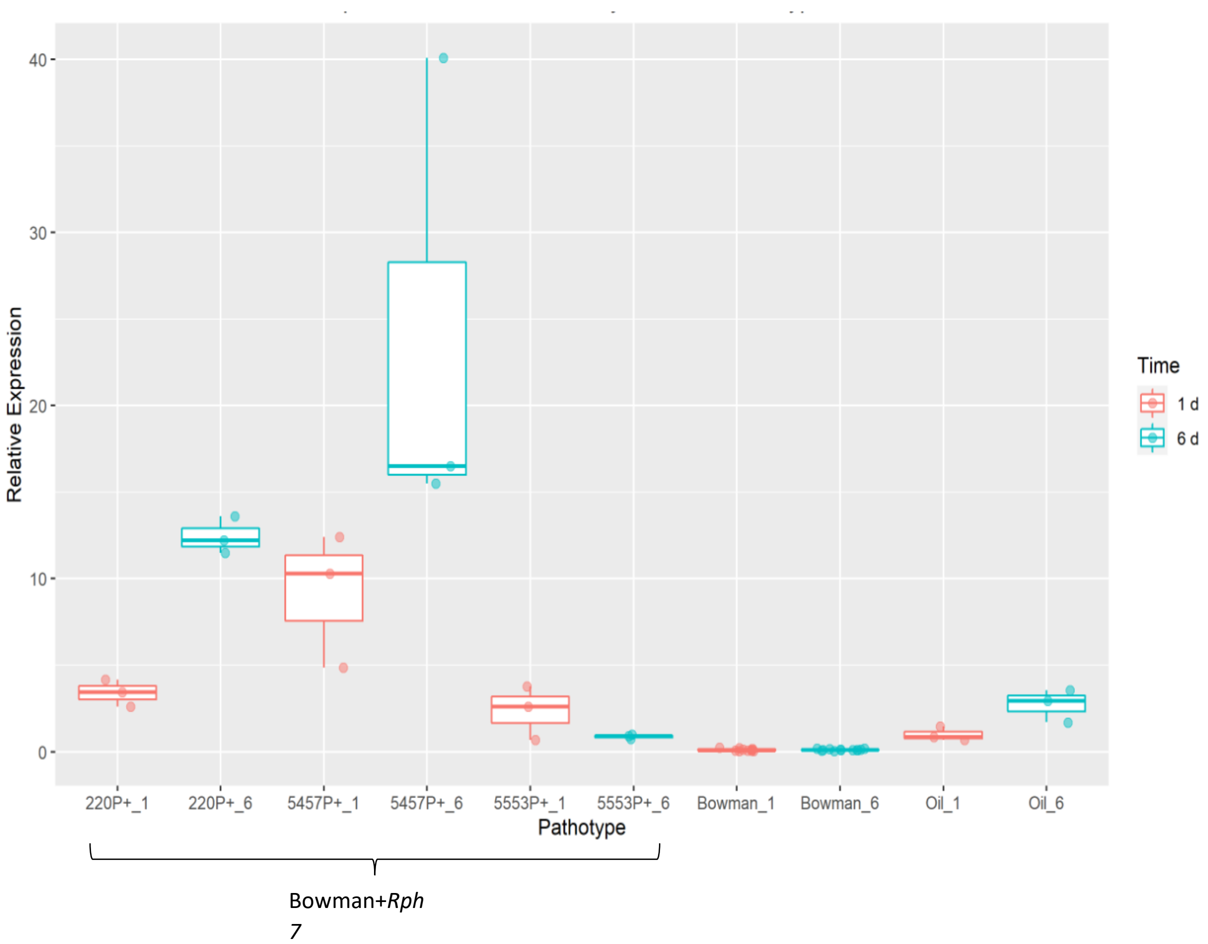

Supplementary Figure 5. Transcript levels of *Rph7* detected by RT-qPCR on days one and six post inoculation in response to virulent and avirulent *Puccinia hordei* pathotypes. The *Rph7* gene in Bowman+*Rph7* (BW758) was upregulated 6 days post infection (dpi) compared with the 1 dpi treatment where only moderate up-regulation was observed relative to the leaf rust susceptible control Bowman when infected by *Rph7*-avirulent *P. hordei* pathotypes (220 P+, and 5457 P+), whereas the transcript levels were unchanged when seedlings were infected by *Rph7*- virulent pathotype (5553 P+). The relative expressions of all samples were compared to the expression of BW758 one dpi after oil treatment which was normalised to 1 and the barley actin gene was used as internal normaliser control. The transcript levels of *Rph7* in un-inoculated seedlings (mock inoculation) is depicted by 'oil'. The data presented captures biological replication (n=3) where the box encompasses two middle quartiles, with central line showing mean. Whiskers extend to the furthest data point within 1.5 times the interquartile range. Source data are provided as a Source Data file.

a

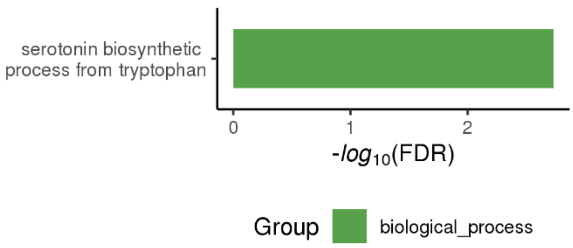

b

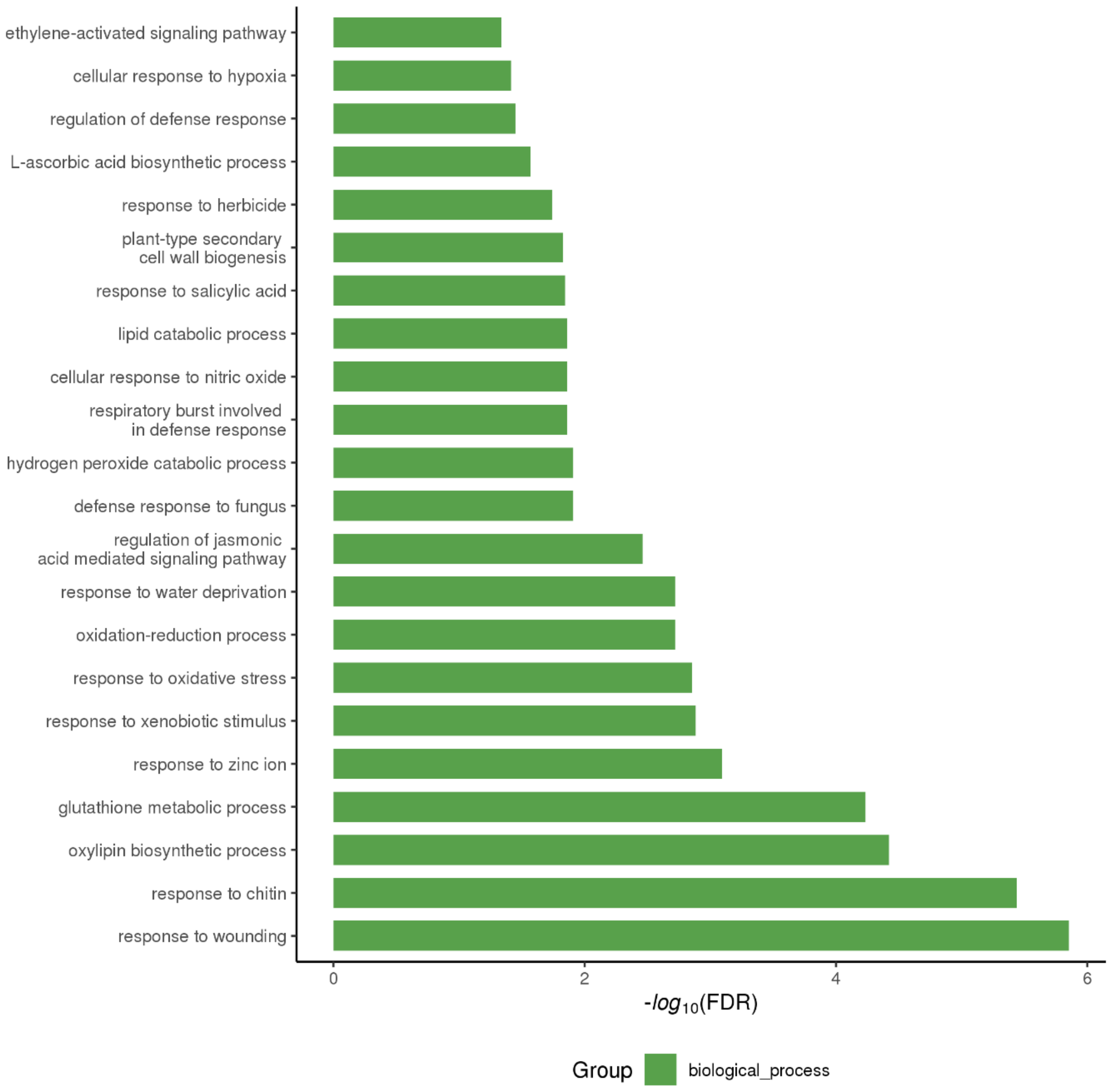

Supplementary Figure 6. Gene ontology enrichment analysis of detected differential expressed genes (DEG) between Bowman (susceptible) vs BW758 (resistant) at day 1 (a) and day 6 (b) after inoculation with *Rph7*-avirulent *Puccinia hordei* pathotype 5457 P+. Overview of Biological Processes detected in DEGs using Triticeae Gene Tribe (http://wheat.cau.edu.cn/TGT/).

Gene	log2FoldChange	Description	
HORVU.MOREX.r3.1HG0000480	11.5167168	Jasmonate-induced protein	
HORVU.MOREX.r3.2HG0205090	8.957075061	Jasmonate-induced protein	
HORVU.MOREX.r3.1HG0000390	8.798539053	Jasmonate-induced protein	
HORVU.MOREX.r3.3HG0330760	8.368007039	Jasmonate-induced protein	
HORVU.MOREX.r3.3HG0330600	7.945613979	Jasmonate-induced protein	
HORVU.MOREX.r3.1HG0000450	7.481327173	Jasmonate induced protein	
HORVU.MOREX.r3.3HG0330720	6.079099777	Jasmonate-induced protein	
HORVU.MOREX.r3.1HG0000470	5.3668004	Jasmonate induced protein	Jasmonate-related
HORVU.MOREX.r3.3HG0330650	5.336017871	Jasmonate induced protein	
HORVU.MOREX.r3.1HG0000430	5.03548161	Jasmonate-induced protein	
HORVU.MOREX.r3.3HG0330620	4.561429708	Jasmonate-induced protein	
HORVU.MOREX.r3.2HG0163880	2.744873093	Jasmonate zim-domain protein	
HORVU.MOREX.r3.4HG0405230	3.641368552	Jasmonate ZIM domain protein	
HORVU.MOREX.r3.5HG0480600	2.450071629	Jasmonate zim-domain protein	
HORVU.MOREX.r3.7HG0669350	-2.248440698	Jasmonate zim-domain protein	
HORVU.MOREX.r3.7HG0669360	-3.843135333	Jasmonate zim-domain protein	
HORVU.MOREX.r3.5HG0519340	7.849094936	Pathogenesis-related protein 1	
HORVU.MOREX.r3.6HG0619530	3.726234428	Pathogenesis-related protein PR-4	
HORVU.MOREX.r3.7HG0662370	2.811237119	Pathogenesis-related protein 1	
HORVU.MOREX.r3.4HG0383530	2.535321426	Pathogenesis-related protein 1	
HORVU.MOREX.r3.5HG0423030	2.687585048	Thaumatin-like protein	Pathogenesis-related
HORVU.MOREX.r3.5HG0423110	2.23607062	Thaumatin-like protein	
HORVU.MOREX.r3.5HG0423060	2.098756469	Thaumatin-like protein	
HORVU.MOREX.r3.3HG0307860	9.475069647	Salicylate O-methyltransferase	
HORVU.MOREX.r3.3HG0297450	5.927803876	WRKY transcription factor	
HORVU.MOREX.r3.5HG0464240	5.622365016	WRKY transcription factor	
HORVU.MOREX.r3.3HG0237360	5.428512893	WRKY family transcription factor	
HORVU.MOREX.r3.1HG0070910	4.086371185	WRKY transcription factor	
HORVU.MOREX.r3.6HG0618760	3.920601689	WRKY transcription factor, putative	
HORVU.MOREX.r3.7HG0710560	2.86203917	WRKY transcription factor	
HORVU.MOREX.r3.1HG0088760	2.84158887	WRKY transcription factor	
HORVU.MOREX.r3.1HG0092420	2.575085338	WRKY transcription factor	WRKY transcription factors
HORVU.MOREX.r3.1HG0070890	2.430739479	WRKY family transcription factor	
HORVU.MOREX.r3.3HG0286660	2.227980799	WRKY transcription factor	
HORVU.MOREX.r3.3HG0273660	2.08655692	WRKY transcription factor	
HORVU.MOREX.r3.7HG0743280	-2.521453205	WRKY transcription factor	
HORVU.MOREX.r3.7HG0743270	-2.649562839	WRKY transcription factor	
HORVU.MOREX.r3.1HG0080940	-2.717058589	WRKY transcription factor	
HORVU.MOREX.r3.4HG0333220	-5.75234814	WRKY transcription factor	
HORVU.MOREX.r3.3HG0222490	5.583724968	Disease resistance protein RPM1	
HORVU.MOREX.r3.2HG0214310	5.499532862	Disease resistance protein RPM1	
HORVU.MOREX.r3.3HG0316460	4.21074174	Disease resistance protein (NBS-LRR class) family	
HORVU.MOREX.r3.2HG0106240	4.030136776	disease resistance protein (TIR-NBS-LRR class)	
HORVU.MOREX.r3.5HG0514590	3.341479475	NBS-LRR disease resistance protein, putative	
HORVU.MOREX.r3.6HG0611400	2.872707076	NB-ARC domain-containing disease resistance protein	
HORVU.MOREX.r3.6HG0630620	2.853213892	Disease resistance protein RGA2	
HORVU.MOREX.r3.7HG0744510	2.29995067	Disease resistance protein (NBS-LRR class) family	Disease resistance proteins
HORVU.MOREX.r3.6HG0559440	-2.154378054	Disease resistance protein RPP13	
HORVU.MOREX.r3.7HG0712390	-2.270475687	NBS-LRR disease resistance protein	
HORVU.MOREX.r3.7HG0639110	-2.630017639	Disease resistance protein (NBS-LRR class) family	
HORVU.MOREX.r3.5HG0489840	-3.103512187	Disease resistance protein RPP13	

Supplementary Figure 7. Subset of day 6 differential expressed genes (DEGs) indicating *ZnF-BED1* (*Rph7*) is likely involved in activating the basal disease response. DEGs between Bowman and BW785 are colour coded according to log2 Fold Change value for upregulated genes in red and downregulated genes in blue. Gene descriptions have been extracted from Morex_V3 (Jul 2020) annotation file.
