# Supplementary material for: A pathogen-induced putative NAC transcription factor mediates leaf rust resistance in barley
Source: Nat Commun. 2023 Sep 6;14:5468. doi: 10.1038/s41467-023-41021-2 (PMC10482968; doi:10.1038/s41467-023-41021-2)
Supplement: Supplementary file 3 — Reporting Summary [file 41467_2023_41021_MOESM3_ESM.pdf]

nature portfolio

Corresponding author(s): Evans Lagudah, Robert Park, Peter Dracatos

Last updated by author(s): Aug 11, 2023

Reporting Summary

Nature Portfolio wishes to improve the reproducibility of the work that we publish. This form provides structure for consistency and transparency in reporting. For further information on Nature Portfolio policies, see our Editorial Policies and the Editorial Policy Checklist.

Statistics

For all statistical analyses, confirm that the following items are present in the figure legend, table legend, main text, or Methods section.

n/a	Confirmed	
☐	☒	The exact sample size (*n*) for each experimental group/condition, given as a discrete number and unit of measurement
☐	☒	A statement on whether measurements were taken from distinct samples or whether the same sample was measured repeatedly
☐	☒	The statistical test(s) used AND whether they are one- or two-sided<br>*Only common tests should be described solely by name; describe more complex techniques in the Methods section.*
☒	☐	A description of all covariates tested
☒	☐	A description of any assumptions or corrections, such as tests of normality and adjustment for multiple comparisons
☐	☒	A full description of the statistical parameters including central tendency (e.g. means) or other basic estimates (e.g. regression coefficient) AND variation (e.g. standard deviation) or associated estimates of uncertainty (e.g. confidence intervals)
☐	☒	For null hypothesis testing, the test statistic (e.g. *F*, *t*, *r*) with confidence intervals, effect sizes, degrees of freedom and *P* value noted<br>*Give P values as exact values whenever suitable.*
☒	☐	For Bayesian analysis, information on the choice of priors and Markov chain Monte Carlo settings
☒	☐	For hierarchical and complex designs, identification of the appropriate level for tests and full reporting of outcomes
☒	☐	Estimates of effect sizes (e.g. Cohen's *d*, Pearson's *r*), indicating how they were calculated

Our web collection on statistics for biologists contains articles on many of the points above.

Software and code

Policy information about availability of computer code

Data collection	http://github.com/streuernb/MutChromSeq, http://github.com/trinityrnaseq/trinityrnaseq, http://github.com/deweylab/RSEM/releases, http://github.com/samtools/samtools/
Data analysis	bwa v0.7.17, SAMtools v1.9 and 1.12, Tophat 2.1.1, Bowtie2 2.4.4, Trinity 2.13.2, ht-seq-count, Google Deepmind's Alphafold2, Pymol UCSF ChimeraX.

For manuscripts utilizing custom algorithms or software that are central to the research but not yet described in published literature, software must be made available to editors and reviewers. We strongly encourage code deposition in a community repository (e.g. GitHub). See the Nature Portfolio guidelines for submitting code & software for further information.

Data

Policy information about availability of data

All manuscripts must include a data availability statement. This statement should provide the following information, where applicable:

- Accession codes, unique identifiers, or web links for publicly available datasets
- A description of any restrictions on data availability
- For clinical datasets or third party data, please ensure that the statement adheres to our policy

http://wheat.cau.edu.cn/TGT/. Rph7 mRNA added to NCBI under accession ORO83044 (http://www.ncbi.nlm.nih.gov/nuccore/ORO83044.1). RNA-Seq data deposited to NCBI SRA Bioproject PRJNA924522 (http://www.ncbi.nlm.nih.gov/bioproject/?term=PRJNA924522). MutChromSeq data submitted ro NCBI SRA archive BioProject ID PRJNA906712 (http://www.ncbi.nlm.nih.gov/bioproject/?term=rph7).

Research involving human participants, their data, or biological material

Policy information about studies with human participants or human data. See also policy information about sex, gender (identity/presentation), and sexual orientation and race, ethnicity and racism.

Reporting on sex and gender	N/A
Reporting on race, ethnicity, or other socially relevant groupings	N/A
Population characteristics	N/A
Recruitment	N/A
Ethics oversight	N/A

Note that full information on the approval of the study protocol must also be provided in the manuscript.

Field-specific reporting

Please select the one below that is the best fit for your research. If you are not sure, read the appropriate sections before making your selection.

[x] Life sciences [ ] Behavioural & social sciences [ ] Ecological, evolutionary & environmental sciences

For a reference copy of the document with all sections, see nature.com/documents/nr-reporting-summary-flat.pdf

Life sciences study design

All studies must disclose on these points even when the disclosure is negative.

Sample size	Three biological replicates (n=3) for each treatment were used for RNA-Seq and qRT-PCR expression which has been well documented as best practice for gene expression studies. For fine mapping we used 1100 F2 generation seeds (2200 gametes) as this was the number that was available based on seed production from F1 plants. Furthermore this number of gametes was sufficient to identify sufficient recombination to eliminate the involvement of 3 of the 4 candidate genes.
Data exclusions	No data was excluded from the analysis or the manuscript.
Replication	For RNA-Seq and qRT-PCR there were three technical replicates sampled for all three of the biological replicates and all attempts were successful. 8-10 biological replicates were used so 8 - 10 different seeds for each barley genotype (mutant/transgenic/Rph7 source) were used for each experiment. The MutChromSeq experiment was repeated twice by independent researchers from two different labs, both ending up with the same candidate gene and mutant positions.
Randomization	Randomisation was applied to the greenhouse and tray positions of the plants grown in all phenotyping experiments to account for environmental variation.
Blinding	Blinding was performed for genotyping of F2 individuals, mutants or transgenic lines where the scientist performing the test did not know the identity or the phenotype of the sample.

Reporting for specific materials, systems and methods

We require information from authors about some types of materials, experimental systems and methods used in many studies. Here, indicate whether each material, system or method listed is relevant to your study. If you are not sure if a list item applies to your research, read the appropriate section before selecting a response.

Materials & experimental systems

n/a	Involved in the study	
[ ]	[x]	Antibodies
[x]	[ ]	Eukaryotic cell lines
[x]	[ ]	Palaeontology and archaeology
[x]	[ ]	Animals and other organisms
[x]	[ ]	Clinical data
[x]	[ ]	Dual use research of concern
[ ]	[x]	Plants

Methods

n/a	Involved in the study	
[x]	[ ]	ChIP-seq
[x]	[ ]	Flow cytometry
[x]	[ ]	MRI-based neuroimaging

Antibodies

Antibodies used	anti-GFP antibody. (mouse IgG1, clones 7.1 and 13.1) 1:2000 dilution
Validation	Website http://www.sigmaaldrich.com/AU/en/product/roche/11814460001, Anti-GFP is tested for functionality and purity relative to a reference standard to confirm the quality of each new reagent preparation. Anti-GFP mouse monoclonal antibodies are >95% pure as determined by SDS-PAGE and ion exchange HPLC analyses.

Dual use research of concern

Policy information about dual use research of concern

Hazards

Could the accidental, deliberate or reckless misuse of agents or technologies generated in the work, or the application of information presented in the manuscript, pose a threat to:

No	Yes	
☒	☐	Public health
☒	☐	National security
☒	☐	Crops and/or livestock
☒	☐	Ecosystems
☒	☐	Any other significant area

Experiments of concern

Does the work involve any of these experiments of concern:

No	Yes	
☒	☐	Demonstrate how to render a vaccine ineffective
☒	☐	Confer resistance to therapeutically useful antibiotics or antiviral agents
☒	☐	Enhance the virulence of a pathogen or render a nonpathogen virulent
☒	☐	Increase transmissibility of a pathogen
☒	☐	Alter the host range of a pathogen
☒	☐	Enable evasion of diagnostic/detection modalities
☒	☐	Enable the weaponization of a biological agent or toxin
☒	☐	Any other potentially harmful combination of experiments and agents
